# Electrochemical Sensor Based on Porous Biochar-Loaded MnO_2_ Nanocomposite for Sensitive Detection of Cd(II)

**DOI:** 10.3390/foods14244252

**Published:** 2025-12-10

**Authors:** Baoli Wang, Kai Tang, Feng Yang

**Affiliations:** 1 Haikou Key Laboratory of Marine Contaminants Monitoring Innovation and Application, Haikou Marine Geological Survey Center, China Geological Survey, Haikou 571127, China; 2 Key Laboratory of Monitoring for Heavy Metal Pollutants, Ministry of Ecology and Environment, Changsha 410019, China; 3 Hainan Engineering Research Center of Tropical Ocean Advanced Opto-Electrical Functional Materials, Haikou 571158, China

**Keywords:** Cd^2+^ electrochemical sensor, manganese dioxide, porous carbon, food safety monitoring

## Abstract

Understanding the extent of cadmium (Cd) contamination and its impact on the food industry and ecosystems is crucial; to this end, electrochemical methods provide sensitive and rapid responses for the in situ detection of heavy metal ions. In this paper, a sensitive Cd^2+^ electrochemical sensor was constructed by using a composite material composed of biomass-derived porous carbon and manganese dioxide (MnO_2_/KLSC). The composite was demonstrated to have a porous structure with quasi-cubic MnO_2_ particles evenly distributed on the carbon matrix. Using MnO_2_/KLSC as the electrode material, a simple electrochemical sensing platform was fabricated for the detection of Cd^2+^. The electrochemical analysis showed that the Cd^2+^ sensor has good performance, with a linear detection range of 0.01–80.0 μmol/L and a detection limit of 9.8 nmol/L. Furthermore, the sensor shows excellent repeatability, reproducibility, and good anti-interference. The sensor was successfully applied to analyze rice and sea water samples, demonstrating satisfactory recovery rates. The good performance of MnO_2_/KLSC/GCE can be attributed to the excellent electrical conductivity, enlarged active surface area, and good electron transfer capability.

## 1. Introduction

With the development of industry, water pollution has become increasingly prominent, particularly heavy metal contamination, which has emerged as a global concern [1,2,3]. Among various heavy metals, cadmium ions (Cd^2+^) have become a highly concerning pollutant due to their high toxicity, degradation difficulty, and bioaccumulation [4,5]. Industrial wastewater discharge, mining operations, electronic waste disposal, and the use of cadmium-containing fertilizers have caused Cd^2+^ to enter the environment and the food chain through various pathways, ultimately endangering human health [6,7]. The half-life of cadmium in the human body ranges from ten to thirty years. Long-term accumulation can cause serious damage to the kidneys, bones, and respiratory system, and can also trigger public health hazards such as “spondylitis” and even be classified as a type of human carcinogen [8]. The World Health Organization (WHO) has listed Cd^2+^ as a priority pollutant for control, establishing strict emission standards [9,10]. However, traditional detection methods, like atomic absorption spectrometry [11], atomic fluorescence spectrometry, [12], X-ray fluorescence spectrometry [13], and inductively coupled plasma mass spectrometry [14], while highly sensitive, suffer from limitations including expensive equipment, complex operations, and cumbersome pretreatment processes, making them inadequate for rapid on-site testing requirements. These limitations have prompted researchers to focus on developing new detection technologies to satisfy the needs for rapid and on-site testing. Electrochemical sensing technology, due to its facile operation, quick determination, low cost, high sensitivity, and ease of achieving online and on-site analysis, has shown prospective potential for application in the field of heavy metal detection in recent years [15,16,17,18,19].

Improving the detection performance of electrochemical sensors is a complex engineering task that involves multiple factors such as functional electrode-sensitive materials synthesis, high-activity electrode interface construction, selection and optimization of recognition component, and system integration and data processing [20,21]. In recent years, nanomaterials have provided new opportunities for sensor technology development because of their particular physicochemical properties, such as high specific surface area, excellent electron transport capability, and surface modifiability [22,23,24]. Sensors constructed from nanomaterials can achieve specific binding with target pollutants through surface functionalization, significantly enhancing the detection selectivity and sensitivity. In particular, sensors based on MOFs, carbon-based materials, and transition metal oxide have shown great potential in heavy metal ion detection [25,26,27]. For instance, Lalawmpuia et al. [28] synthesized a novel Ti-MOF using a solvothermal method and employed it as an electrode-modifying material to fabricate a sensor for detecting Cd^2+^ and Pb^2+^ simultaneously, yielding the detection limits of 0.59 and 1.02 μg/L for Cd^2+^ and Pb^2+^. Furthermore, the sensor showed good selectivity and was successfully applied in the detection of Cd^2+^ and Pb^2+^ in actual samples. Ajdrai et al. [29] prepared a multiwall carbon nanotubes–poly(2-aminothiophenol)@silver nanoparticles nanocomposite, and it was used as an electrode-modifying material, producing good detection performance for Pb^2+^ and Cd^2+^. The linear response ranges of the constructed sensors for Pb^2+^ and Cd^2+^ are 0.5–60.0 nmol L^−1^ and 8.0–50.0 nmol L^−1^, with detection limits of 0.125 nmol L^−1^ for Pb^2+^ and 1.47 nmol L^−1^ for Cd^2+^, respectively.

Additionally, Wu et al. [30] employed an artificial ZIF-67 template to synthesize a Co_3_O_4_ carbon nanofiber aerogel composite as a modifier for electrochemical sensors. The unique aerogel skeleton provided suitable conditions for the enrichment of heavy metal ions and rapid electron transfer, thus resulting in a high sensitivity, a low detection limit (2.67 μg·L^−1^), and a wide detection range (50–1000 μg·L^−1^) in the detection of Cd^2+^.

While nanosensors have made significant strides in Cd^2+^ detection, practical applications still face challenges. Key research priorities include balancing sensor sensitivity with stability and reducing costs to enable widespread adoption in resource-constrained regions. Biomass-derived carbon exhibits significant advantages in the electrodes of electrochemical sensors. Its high specific surface area and porous structure provide ample active sites for substance adsorption and electron transfer, effectively enhancing the detection sensitivity. The abundant functional groups on the surface facilitate modification, which can enhance the selectivity for specific target substances. At the same time, the raw materials are abundant and inexpensive, and the synthesis procedure is eco-friendly, giving it excellent cost-effectiveness and sustainable development potential. It also has good chemical stability and conductivity, making it a promising material for constructing high-performance and low-cost sensors. At the same time, transition metal oxides are rich in active sites, and they have a strong adsorption capacity and high selectivity for heavy metal ions [30]. The unique electronic structure endows them with excellent electrocatalytic activity, which can significantly enhance the detection signal and improve the sensitivity. The material has good stability, a long service life, and can easily be nano-sized to increase the specific surface area, further improving the detection performance.

**Scheme 1:**
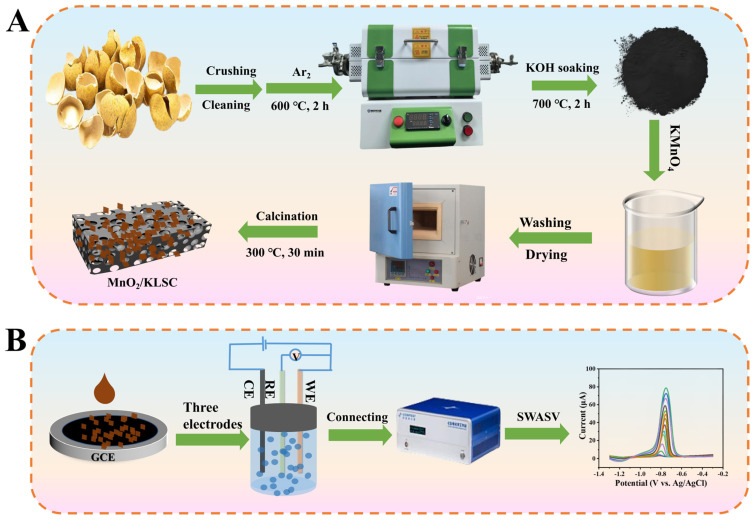
(**A**) Preparation procedure of MnO_2_/KLSC and (**B**) the MnO_2_/KLSC/GCE sensor fabrication for the detection of Cd^2+^.

Based on the above considerations, this study synthesized a biomass-derived carbon loaded MnO_2_ composite (MnO_2_/KLSC) as the electrode modify material, which ingeniously combines the “structural advantages” of biochar (large specific surface area, porous, conductive, and low cost) with the “functional advantages” of MnO_2_ (high capacitance, high catalytic activity). It successfully addresses the key issues faced by a single material in electrochemical sensing applications, such as poor conductivity, few active sites, unstable structure, and high cost. The material preparation and sensor fabrication processes are shown in Scheme 1. The crystal structure and surface topography of the MnO_2_/KLSC were characterized by XRD and TEM. The elemental composition and valence states were studied by EDS mappings and XPS, with the BET specific surface area calculated through N_2_ adsorption–desorption isotherms. Moreover, the electrochemical detection performance of the MnO_2_/KLSC-modified sensor was explored at low concentrations of Cd^2+^ through electrochemical pre-enrichment and anodic-stripping processes. As a result, MnO_2_/KLSC produced an excellent electrochemical sensing performance for Cd^2+^ detection with a low LOD and a wide linear range. This provides a highly promising solution for the development of next-generation high-performance, low-cost, and environmentally friendly electrochemical Cd^2+^ detection sensors.

## 2. Materials and Methods

### 2.1. Reagents

Longan shell was collected from the local market in Haikou. Concentrated hydrochloric acid (HCl, 36%), K_3_Fe(CN)_6_, K_4_Fe(CN)_6_, KCl, KOH, KMnO_4_, HNO_3_, HClO_4_, 5% Nafion ethanol solution, and BR buffer solution were bought from Aladdin (Shanghai, China). All chemical reagents were used as received. The standard solutions of Ca^2+^, Fe^3+^, Fe^2+^, Ni^2+^, Co^2+^, Cu^2+^, Na^+^, K^+^, Mg^2+^, Zn^2+^, and Pb^2+^ were purchased from Tanmo Quality Inspection Technology Co., Ltd. (Beijing, China). The ultrapure water was prepared from the Purifier FST-III-20 water purification system (Shanghai Fushite Instrument Equipment Co., Ltd., China) and used throughout the entire experiment.

### 2.2. Instruments

Transmission electron microscopy (TEM) was carried out using a JEM-2010F microscope (JEOL, Tokyo, Japan) operated at 200 kV. X-ray photoelectron spectroscopy (XPS) was performed on an AXIS HIS 165 spectrophotometer (Kratos Analytical, Manchester, UK). X-ray diffraction (XRD) was carried out by a D/Max-2500 V X-ray diffractometer (Rigaku, Japan) with Cu-Ka1 radiation (λ = 1.5406 Å) in the 2θ range of 20–80°. N_2_ adsorption–desorption analysis was performed on a surface area and porosity analyzer (ASAP 2020, Micromeritics, Norcross, USA), and the specific surface area and the pore size distribution were calculated by using Brunauer–Emmett–Teller (BET) theory and Barrett–Joyner–Halenda (BJH) analysis, respectively. The organic elements in the longan shells were determined by an Elementar Vario EL Cube, and the other elements were determined by ICP-MS (Thermo iCAP-Q, Thermo Fisher Scientific, Waltham, MA, USA).

### 2.3. Synthesis of MnO_2_/KLSC

The remaining pulp and impurities inside the longan shells were removed, and it was then washed with water, dried, and crushed into powder that could pass through an 80-mesh sieve. The raw material identity affects the carbon texture, heteroatom content, and reproducibility. Thus, the elemental content of the raw material was determined, which confirmed that the contents of C, N, H, and S are 41.54%, 0.93%, 5.12%, and 0.02%, respectively. In addition, the material also contains some other elements, including K, P, Ca, Fe, Mg, Na, and Zn, with contents of 0.245%, 0.007%, 0.016%, 0.002%, 0.013%, 0.004%, and 0.0002%, respectively. For the MnO_2_/KLSC preparation, 8.5 g of the obtained powder was put into a crucible and placed in the tube furnace; followed by heating to 600 °C under an Ar atmosphere for 2 h with a programmed temperature rise rate of 5 °C/min to obtain the black powder; and followed by etching with HCl for 24 h, washing, and drying. HCl can effectively remove the metal elements from the obtained product, making it easier to study the structure and composition of the intended product in the subsequent research. The obtained substance was then mixed with 10 mL of 5 mol/L KOH solution and continuously stirred at 70 °C until the liquid completely evaporated. The obtained substance was carbonized at 700 °C for 2 h, resulting in the product KLSC. Finally, 3 g KLSC and 0.5 g KMnO_4_ were dispersed in 30 mL ultrapure water, stirred for 5 h, and followed by filtering and drying. The resulting solid was then put into a muffle furnace and sintered at 350 °C for 30 min to obtain the intended material (labeled as MnO_2_/KLSC). The preparation procedure of MnO_2_/KLSC is shown in Scheme 1A.

### 2.4. Fabrication of MnO_2_/KLSC Sensor

The surface of the GCE was first polished and cleaned ultrasonically for use. Then 2.0 mg of MnO_2_/KLSC was dispersed in 1 mL ultrapure water and ultrasonically treated for 1 h to obtain a uniform 2.0 mg/mL suspension. Then 6.0 μL of the suspension was dropped onto a GCE surface and dried in air at ambient temperature. Subsequently, 5.0 μL 0.5% Nafion was dripped on the electrode surface and dried in air to obtain the modified electrode (MnO_2_/KLSC/GCE). For comparison, the KLSC/GCE electrode was prepared by adopting KLSC as the modified material under the same experimental conditions. The sensor fabrication process is shown in Scheme 1B.

### 2.5. Electrochemical Investigations

All electrochemical experiments were carried out on a CHI660E electrochemical workstation using a standard three-electrode system, where the working electrode was MnO_2_/KLSC/GCE, reference electrode was Ag/AgCl (saturated KCl), and the counter electrode was platinum. Cyclic voltammetry (CV) was tested in the potential range of 0.3–0.7 V with a scan rate of 100 mV/s. When testing at different scan rates, it was fit to 10–120 mV/s. For square wave stripping voltammetry (SWASV), the potential window was set to −1.3 V and −0.3 V, with an amplitude of 25 mV, a potential increment of 4 mV, a frequency of 15 Hz, and a quiet time of 2.0 s. Each measurement was repeated 5 times. The chosen electrolyte in the electrochemical testing experiment was 0.04 mol/L BR (pH = 5), and its pH was adjusted using 0.1 mol/L NaOH solution.

### 2.6. Detection of Cd^2+^ in Rice and Sea Water

Rice was purchased from a local farmers’ market, and seawater was collected from Xiu Ying Port in Haikou. Seawater samples were prepared as follows: first, 50 mL of the sample was filtered through 0.45 μm and 0.2 μm filter membranes in turn. Following centrifugation, the supernatant was gathered and 100 μL of it was mixed with 1 mL BR (pH = 5) solution for a real sample analysis under ideal circumstances. For the rice samples, first, 10 g of rice was ground into powder, and a 10 mL mixed solution of HNO_3_ and HClO_4_ (with a volume ratio of 4:1) was prepared. Then the rice powder was added into the above mixed acid solution to form a suspension followed by heating at 150 °C for 6 h to obtain a transparent solution. Subsequently, the pH of the solution was adjusted to 5.0 using 0.1 M NaOH and then analyzed using the SWASV method.

## 3. Results and Discussion

### 3.1. Material Characterizations

**Figure 1:**
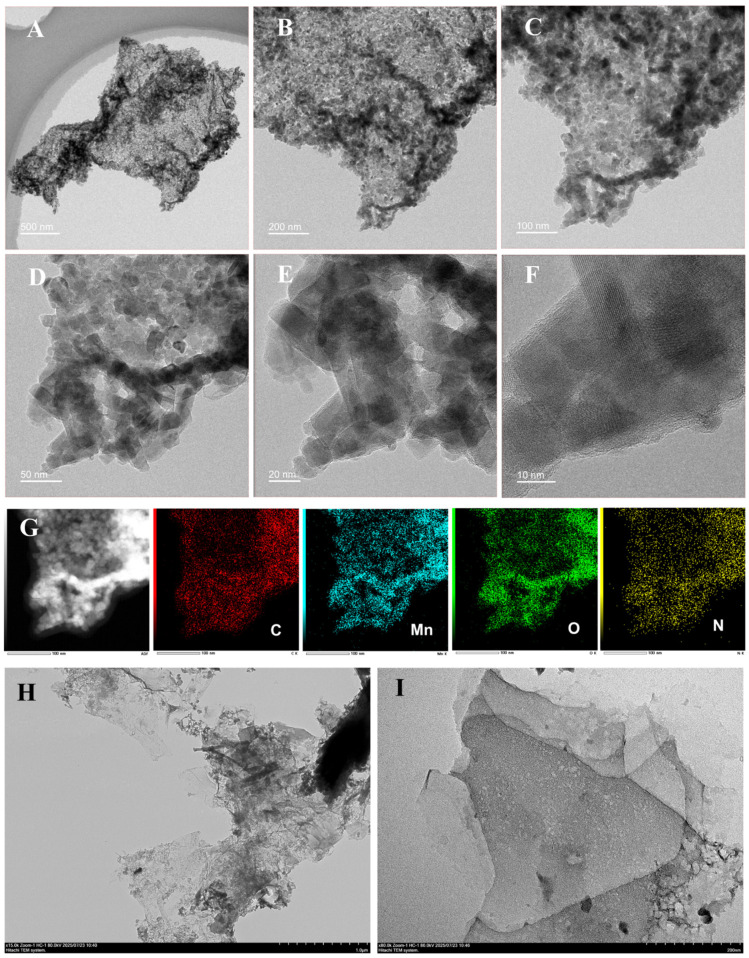
(**A**–**E**) TEM images and (**F**) HRTEM images of MnO_2_/KLSC@Fe_3_C; (**G**) EDS-mapping images of MnO_2_/KLSC; (**H**,**I**) TEM images of KLSC.

In order to investigate the morphology and elemental composition of the material, (HR)TEM and elemental mapping analyses were conducted on the material. As displayed in Figure 1, the TEM images (A–D) show that a dense cluster of square-like nanoparticles with an average size of 12.5 nm are distributed on the carbon matrix. Furthermore, these nanoparticles are distributed very evenly with very few cases of agglomeration. It can be obviously observed that the distance of 0.16 nm exactly represents the crystal plane spacing of the MnO_2_ (211) crystal plane, which confirms that KMnO_4_ was transformed into MnO_2_ during the calcination procedure without the formation of any other manganese oxides (Figure 1F). In the HRTEM, the typical graphite carbon lattice pattern was not observed, which might be due to the relatively low degree of graphitization of the carbon framework or because the dense MnO_2_ nanoparticles cover the lattice patterns. In the EDS mappings (Figure 1G), the main elements including C, Mn, O, and N can be detected and are uniformly distributed in MnO_2_/KLSC, where the O element mainly originates from the calcination process in the air and a small amount of N element comes from the biomass raw material. Furthermore, the morphology of KLSC was also analyzed. As shown in Figure 1H, it can be seen that the KLSC presents a fragmented structure within the micrometer scale range. As the magnification increases (Figure 1I), a distinct sheet structure can be observed and there are obvious holes on the surface of the lamina, which can be attributed to the corrosive effect of KOH, further increasing the specific surface area of KLSC. The porous structure and larger specific surface area provides more physical sites for the loading of MnO_2_, thereby increasing the active sites of the composite material and offering more abundant channels for the transport of substances and electrons during the electrochemical process [30].

**Figure 2:**
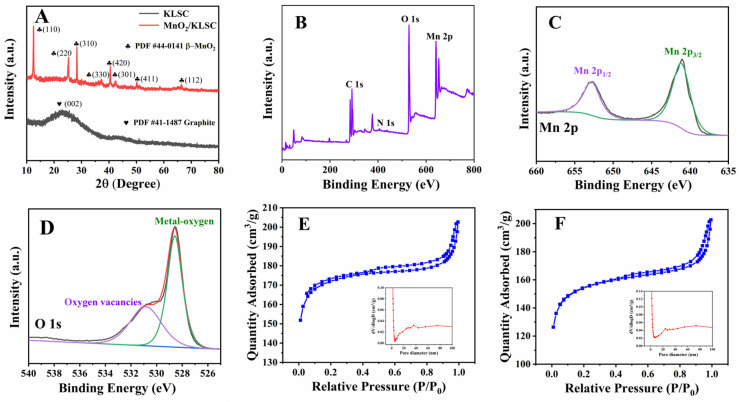
(**A**) XRD patterns of KLSC and MnO_2_/KLSC; (**B**) XPS spectrum of MnO_2_/KLSC; (**C**,**D**) fine diluted Mn 2p and O1s XPS spectra of MnO_2_/KLSC; (**E**,**F**) nitrogen adsorption–desorption isotherms of KLSC and MnO_2_/KLSC (the insert images are the corresponding pore diameter distribution curves).

XRD was conducted to study the crystal phase and composition, with the results depicted in Figure 2A. It is obvious that two wide and broad diffraction peaks located at about 25.4° and 42.1° were observed, which can be attributed to the typical peaks of graphite carbon. The weaker of the peak intensities can be assigned to the lower carbonation temperature and without adding the graphitized catalyst. As for MnO_2_/KLSC, a set of peaks representing MnO_2_ can obviously be detected, where the peaks at 12.8°, 25.7°, 28.8°, 37.5°, 41.2°, 42.0°, 49.8°, and 66.7° correspond to the (110), (220), (310), (330), (420), (301), (411), and (112) crystal planes of MnO_2_ (PDF#44-0141). This confirms that the KMnO_4_ converted to MnO_2_ in the calcination process, which is consistent with the HRTEM results.

Furthermore, XPS analysis was carried out to study the elemental composition and electronic properties of MnO_2_/KLSC. As shown in Figure 2B, it clearly confirms the coexistence of the characteristic C, Mn, O, and N elements in MnO_2_/KLSC. The fine-defined Mn 2p spectra (Figure 2C) revealed spin energy separations of 11.57 eV between Mn 2p_3/2_ (642.7 eV) and Mn 2p_1/2_ (654.3 eV), corresponding to the Mn^4+^ and Mn^3+^ oxidation states, which is consistent with previously reported values [31]. Thereafter, for O 1 s, the deconvoluted XPS spectrum depicted in Figure 2D presents the peaks at 529.8 eV and 530.5 eV, attributed to surface oxygen vacancies (Ovs) and metal–oxygen bonds (Metal-Os), respectively [32]. Based on literature reports, this high oxygen vacancy content is expected to enhance the catalytic performance of the MnO_2_ [33]. The N_2_ adsorption–desorption isotherms of KLSC and MnO_2_/KLSC both display a type IV isotherm, revealing the presence of mesopores (Figure 2E,F). The BET surface areas of KLSC and MnO_2_/KLSCs were calculated as 678.53 m^2^/g and 596.90 m^2^/g, respectively. The large specific surface area of KLSC is due to the fact that when it is mixed with KOH, KOH etches away some of the amorphous C and C at structural defects in the carbon framework, creating new pore structures, thereby increasing the specific surface area. Meanwhile, the specific surface area of MnO_2_/KLSC is smaller than that of KLSC, which may have been because some of the pore structures of the carbon framework were occupied by the particles after loading MnO_2_. This indicates that there was a slight reduction in the specific surface area after loading MnO_2_, but it did not cause significant changes to the surface structure. The pore size distribution curve (insert of Figure 2E,F) shows that the pore diameters of KLSC are mainly concentrated around 2 nm and 40 nm, while the pore diameters of the composite are mainly around 2 nm and 30 nm, which might be because the particle accumulation causes the pores of around 40 nm to become smaller after loading MnO_2_ nanoparticles.

### 3.2. Electrochemical Behaviors of Different Electrodes

**Figure 3:**
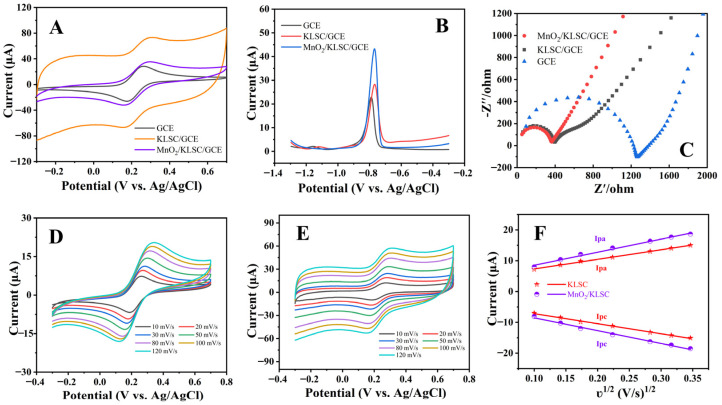
(**A**) CV curves of bare GCE, KLSC/GCE, and MnO_2_/KLSC/GCE in 1.0 mmol/L [Fe(CN)_6_]^2+/3+^ and 0.5 mol/L KCl solution; (**B**) SWASV curves and (**C**) EIS spectra of GCE, KLSC/GCE, and MnO_2_/KLSC/GCE in 0.04 M BR (pH = 5) containing 10 µM Cd^2+^; CV curves of (**D**) MnO_2_/KLSC/GCE and (**E**) KLSC/GCE in a solution of 1.0 mmol/L [Fe(CN)_6_]^2+/3+^ and 0.5 mol/L KCl solution at different scanning rates (10−120 mV/s); (**F**) corresponding linear plots of the peak current versus the square root of scanning rate (ʋ^1/2^).

The electrochemical behaviors of the bare GCE, KLSC/GCE, and MnO_2_/KLSC/GCE were first investigated by CV and EIS in 1.0 mmol/L [Fe(CN)_6_]^2+/3+^ and 0.5 mol/L KCl. The bare GCE showed a pair of well-defined and reversible redox peaks (Figure 3A). The KLSC/GCE presented a greater redox performance, implying the enhanced electron transfer at the surface of the electrode. The loading of MnO_2_ onto the KLSC skeleton further enhanced the electrochemical response. Furthermore, the SWASV curves for the three electrodes in the 0.04 M BR (pH = 5) electrolyte solution containing 10 μmol Cd^2+^ were collected. As depicted in Figure 3C, the bare GCE has the smallest peak current, and MnO_2_/KLSC/GCE has the biggest peak current, with the value of 45.6 μA. The peak current of KLSC/GCE is significantly lower than that of MnO_2_/KLSC/GCE, which might be because the loading of MnO_2_ enhanced the catalytic activity of the material. Furthermore, the peak voltages of KLSC/GCE and MnO_2_/KLSC/GCE shifted to the right compared with GCE, which might have been due to the loading of the materials. As depicted in Figure 3C, the charge transfer resistance values of MnO_2_/KLSC/GCE were found to be smaller than those of GCE and KLSC/GCE, which disclosed that the loaded MnO_2_ effectively reduced the charge transfer resistance, leading to the best conductivity and fastest charge transfer kinetics. The electrochemical active surface area (ECSA) was investigated using CV by varying the scan rates from 10 to 120 mV/s (Figure 3D,E). The ECSAs were calculated using the Randles–Sevcik equation [34], yielding values of 0.126 cm^2^ (KLSC/GCE) and 0.171 cm^2^ (MnO_2_/KLSC/GCE) based on the linear equation between the peak current and ʋ^1/2^ (Figure 3F). Obviously, the MnO_2_/KLSC/GCE has a comparatively large ECSA, which is conducive to promoting the ion adsorption and subsequent electrochemical reactions.

**Figure 4:**
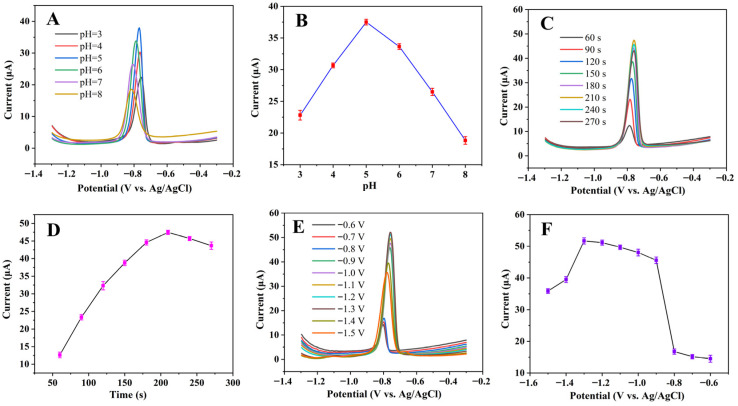
(**A**) SWASV curves of 10 μmol/L Cd^2+^ on MnO_2_/KLSC/GCE in 0.04 M BR with pH ranging from 3.0 to 8.0; (**B**) Ipa versus pH; (**C**) SWASV curves of 10 μmol/L Cd^2+^ on MnO_2_/KLSC/GCE in 0.04 M BR pH = 5 with different deposited time; (**D**) Ipa versus deposited time; (**E**) SWASV curves of 10 μmol/L Cd^2+^ on MnO_2_/KLSC/GCE in 0.04 M BR pH = 5 with different deposited potential; (**F**) deposited potential versus peak current.

The pH value of BR, deposition time, and deposition potential in the electrochemical test conditions were first optimized, with the results displayed in Figure 4A–F. The peak current first increases and then decreases in the pH range of 3 to 8. The maximum current was obtained at pH 5.0. When the deposition time increased from 60 s to 210 s, the peak current increased. However, when the deposition time continued to increase to 270 s, the peak current decreased instead. For the deposition potential, from −0.6 V to −0.9 V, the peak current increased sharply. While between −0.9 V and −1.3 V, the peak current became relatively stable; it reached the maximum current at −1.3 V. When the deposition potential was greater than −1.3 V, the peak current dropped sharply. Thus, the optimal pH was 5.0, and the optimal deposition time and deposition potential were 210 s and −1.3 V, respectively. All the subsequent tests were conducted under the optimal experimental conditions.

### 3.3. Analytical Performance of LVX at LSC@Fe_3_C/GCE

**Figure 5:**
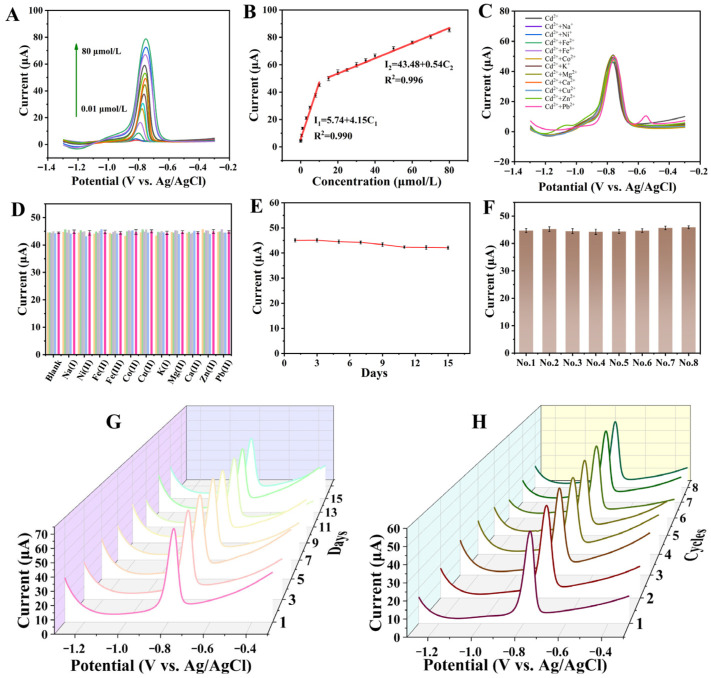
(**A**) SWASV curves of Cd^2+^ on MnO_2_/KLSC/GCE in 0.04 M BR with the concentration ranging from 0.01 to 80.0 μmol/L; (**B**) Ipa versus Cd^2+^ concentration; (**C**) the SWASV curves of 10 μmol/L Cd^2+^ on MnO_2_/KLSC/GCE in 0.04 M BR containing 100 μmol/L different interfering ion and (**D**) the corresponding peak current response histogram; (**E**) the peak current of 10 μmol/L Cd^2+^ on MnO_2_/KLSC/GCE in 0.04 M BR pH = 5 on different days (n = 5) and (**G**) the SWASV curves; (**F**) the peak current of one single electrode (n = 8) used 8 consecutive times and (**H**) the SWASV curves.

The SWASV method was employed to evaluate the current responses to varying standard concentrations of Cd^2+^. As displayed in Figure 5A,B, the peak current increases when the Cd^2+^ concentration increases. The linear range can be divided into two parts: I_1_ (μA) = 5.74 + 4.15C_1_ (μmol/L) (n = 5, R^2^ = 0.990) and I_2_ (μA) = 43.48 + 0.54C_2_ (μmol/L) (n = 5, R^2^ = 0.996). The LOD is defined as 3σ/S, where σ denotes the standard deviation and S is the slope of the linear regression equation. The LOD value was then calculated as 9.8 nmol/L.

MnO_2_/KLSC/GCE presents superior detection performance for Cd^2+^ detection compared with some previously reported Cd^2+^ sensors, as listed in Table 1, exhibiting a higher sensitivity, a broader detection range, and a lower detection limit. The reasons for the performance enhancement might be (i) the large specific surface area of the material provides affluent channels for the transport of substances and electrons, (ii) the increased exposure of Mn^4+^/Mn^3+^ provided more catalytic active sites, and (iii) the synergistic effect between the KLSC carbon matrix and the uniformly dispersed MnO_2_ nanoparticles.

### 3.4. Stability, Repeatability, Reproducibility, and Interference Study

The effectiveness of the constructed sensor at detecting Cd^2+^ in the presence of other interfering ions commonly present in real water samples was further investigated. Eight potential interfering ions commonly found in real samples, including Ca^2+^, Fe^3+^, Fe^2+^, Ni^2+^, Co^2+^, Cu^2+^, Na^+^, K^+^, and Mg^2+^, were chosen. The ability of the proposed sensor to discriminate the target Cd^2+^ from the interferents was investigated by monitoring the changes in the SWASV peak current and potential of 10 µmol/L Cd^2+^. Figure 5C,D shows the SWASV curves and the corresponding histogram observed for 10 µmol/L Cd^2+^ in the presence of interferents (concentrations ratio 1:10). It can be seen that the interfering ions have little effect on the peak current of Cd^2+^. It is worth noting that the peaks of Zn^2+^ (−1.08 V) and Pb^2+^ (−0.56 V) can also be observed, which caused a slight shift in the peak position of Cd^2+^.

The reproducibility and stability of an electrode are also important parameters when assessing the electrode’s properties. The 10 μmol/L Cd^2+^ solution (in 0.04 M BR pH = 5) was chosen as the test sample to investigate the reproducibility and stability. The SWASV curves for the detection of the same concentration of Cd^2+^ solutions using the same electrode at days 1, 3, 5, 7, 9, 11, 13, and 15 were collected (n = 5, RSD < 1.25%). After 15 days, a slight decrease in the peak current of the electrode was detected (Figure 5E,G). The solution was tested eight consecutive times using a single electrode, and the curve changes in each test are very small. As displayed in Figure 5F,H, the response current remains almost constant, disclosing a good repeatability (RSD < 1.77%). During the entire experimental testing process, the electrodes were stored in a refrigerator at 4 °C with a humidity of 55–60%. The above results verify that the MnO_2_/KLSC/GCE has good reproducibility and stability.

### 3.5. Real Sample Detection

By testing the content of Cd^2+^ in rice and seawater samples, the practical application capability of the constructed sensor was investigated by the standard addition method. The treatment procedure of the samples is detailed in Section 2.6. The test results are listed in Table 2. They reveal that the MnO_2_/KLSC/GCE sensor presents a recovery rate in the range of 97.80–104.60%, which was calculated as less than 4.92%. This result indicates the accuracy of MnO_2_/KLSC/GCE and also confirms its actual sample detection capability. It may provide technical support for food safety supervision and environmental testing.

## 4. Conclusions

This study proposes a high-performance modified electrode based on MnO_2_/KLSC for the determination of Cd^2+^ in rice and sea water samples. The integration of MnO_2_ nanoparticles into a porous biomass-derived carbon skeleton accelerated the electron transfer and facilitated the specific connections with Cd^2+^, further remarkably enhancing its detection performance. Thus, it displayed a wide linear detection range (0.01 µmol/L to 80 µmol/L) and a low detection limit of 9.8 nmol/L, making it adaptive for Cd^2+^ analysis. Furthermore, the sensor also showed good repeatability, reproducibility, and stability, with recovery rates ranging from 97.80% to 104.60%. Moreover, the sensor presented good anti-interference capacity in the presence of Ca^2+^, Fe^3+^, Fe^2+^, Ni^2+^, Co^2+^, Cu^2+^, Na^+^, K^+^, Mg^2+^, Zn^2+^, and Pb^2+^ ions, and showed satisfactory performances regarding rice and seawater samples detection. This sensor proposal underlines the promising potential as an economical and efficient route for safeguarding public health and environmental monitoring.

## Figures and Tables

**Table 1:** Comparisons of the constructed Cd^2+^ sensor with the previously reported Cd^2+^ sensors.

Electrodes	Method	Linear Range (μmol/L)	LOD (nmol/L)	Ref.
Fe_3_O_4_/Bi_2_O_3_/C_3_N_4_/GCE	SWASV	0.01–3	3.0	[35]
g-C_3_N_4_/CuNps	DPASV	0.0089–0.89	0.098	[36]
Co-ZIF@LP/GCE	SWASV	1–10	28	[37]
p(Cys).gCN|GCE	SWV	1–100	4.90	[38]
GO@ZnO@SiO_2_	DPV	0.005–0.0175 0.0175–0.15	12.5	[39]
ZIF-7@PANI/GCE	DPV	0.02–30	10.6	[40]
MnO_x_/CC	DPV	0.1–100	80	[41]
Nano-PPCPE	DPASV	0.1–3.0	78	[42]
Poly(BPE)/g-C_3_N_4_	DPV	0.12–7.2	18	[43]
PVP/GNs@Fe-MOF	LSV	0.03–1.0	10	[44]
ZnCo/N-PC@NACS/GCE	SWV	0.1–80	33	[45]
MnO_2_/KLSC/GCE	SWASV	0.01–80	9.8	This work

**Table 2:** Concentration of Cd^2+^ in rice and sea water determined by standard addition method.

Sample	Detected (μmol/L)	Added (μmol/L)	Found (μmol/L)	Recovery (%)	RSD (%)
Rice	0.00	5	5.46	104.60%	3.36%
		10	10.08	97.80%	4.92%
		20	20.15	101.45%	2.37%
Sea water	0.00	5	5.37	95.40%	4.15%
		10	10.49	104.90%	4.07%
		20	20.13	100.65%	1.88%

## Data Availability

The original contributions presented in this study are included in the article. Further inquiries can be directed to the corresponding author.
